# Characterization of the first chloroplast genome of *Euchresta tubulosa* Dunn and its phylogenetic analysis

**DOI:** 10.1080/23802359.2021.1972866

**Published:** 2021-09-09

**Authors:** Wei Zhuo, Fengming Ren, Liqiang Wang, Shuangkou Chen, Yanbing Chen, Hongyan Huang

**Affiliations:** a Chongqing Key Laboratory of Industrial Fermentation Microorganism, Department of Chemistry and Chemical Engineering, Chongqing University of Science and Technology, Chongqing, China; b Chongqing Institute of Medicinal Plant Cultivation, Research and Utilization on Characteristic Biological Resources of Sichuan and Chongqing Co-construction Lab, Chongqing, China; c College of Pharmacy, Heze University, Shandong, China; d Chongqing Research Institute of Daily-used Chemical Industry, Chongqing, China

**Keywords:** Chloroplast genome, *Euchresta tubulosa*, medicinal plant, phylogenetic analysis, Fabaceae

## Abstract

*Euchresta tubulosa* Dunn not only is a national second-level protected wild plant in China, but also has a long history as a source plant in traditional Chinese medicine. The chloroplast (cp) genome of *E. tubulosa* was 154,102 bp, consisting of a large single-copy region (LSC: 92,877 bp), a small single-copy region (SSC: 36,645 bp), and a pair of inverted repeat regions (IRb and Ira: 12,290 bp, respectively). These sequences encoded 123 genes, including 78 protein-coding genes, 37 tRNA genes, and 8 rRNA genes. The phylogenetic analysis showed that *E. tubulosa* is close to *Lupinus* species.

*Euchresta tubulosa* Dunn, with dried roots known as ‘Shan Dou Gen’ in traditional Chinese medicine, is an evergreen vine-like shrub of the genus *Euchresta* in Fabaceae. *Euchresta tubulosa* is a national second-level protected wild plant in China, which is scattered in parts of Guangdong, Guangxi, Sichuan and Zhejiang of China, as well as in Japan. It not only has the effects of clearing away heat, detoxifying, reducing swelling and pain, but also has the effects of treating enteritis, diarrhea, abdominal pain, and stomach pain. *Euchresta tubulosa* is one of the common Chinese herbal medicines in the folk (Lin et al. 2009; Li et al. 2011). Modern pharmacological studies have found that *E. tubulosa* contains flavonoids (Matsuura et al. 1993), alkaloids (Li et al., 2019), steroids and other compounds (Li et al. 2014). At the same time, *Euchresta* species has the activities of anti-tumor, inducing cell apoptosis, central inhibition, antioxidant (Toda and Shirataki 2006), regulating blood lipids, antibacterial and other pharmacological activities (Li and Li 2016). However, as an important medicinal plant, *E. tubulosa* has not been reported on genome related information. In this study, we report the first complete chloroplast (cp) genome sequence of *E. tubulosa*, which not only helps to better understand its evolution and population genetics, but also provides important information for the phylogeny of *Euchresta*.

Fresh leaves of *E. tubulosa* were collected from Nanchuan, Chongqing, China (107°21′E, 29°14′N, 600 m). The voucher specimen was conserved in Chongqing Institute of Medicinal Plant Cultivation (accession number: CIMPC-RFM-20210403, Contact person: Fengming Ren; Email: 348080877@qq.com). In the experiment, the high-quality whole genomic DNA was extracted by the kit method (Beijing Kinco Biological Company). The purity and integrity of the DNA were analyzed by Nanodrop (Thermo Fisher Scientific) and agarose gel electrophoresis. Total DNA was used to generate libraries with insert size of 350 bp and was generated raw pair-end reads by Illumina Hiseq 2500 Platform (Illumina, Hayward, CA, USA).

Low-quality readings and adapters in the raw data are deleted by trimmomati (version 0.35) with default paprameters (Bolger et al. 2014). About 15 G clean data was obtained. Using the clean data with 150 bp paired-end read lengths obtained from the raw data, a cp genome was assembled by NOVOPlasty (version 4.1) with the default parameters (Nicolas et al. 2016) and annotated by CPGAVAS2 (http://47.96.249.172:16019/analyzer/home) (Shi et al. 2019). Finally, manually correct the start and stop codons of protein-coding genes.

The cp sequence of *E. tubulosa* was submitted to NCBI, and the accession number was MZ323108. The cp genome of *E. tubulosa* is 154,102 bp in size, containing four regions: large single-copy region (LSC: 92,877 bp), small single-copy region (SSC: 36,645 bp), and a pair of inverted repeat regions (IR: 12,290 bp). The GC contents of whole genome and each genomic regions were 36.27% (whole genome), 34.18% (LSC region), 40.12% (SSC region), 38.41% (IR region). A total of 123 genes were annotated, including 78 protein-coding genes, 37 tRNA genes, and 8 rRNA genes.

To investigate its taxonomic status, additional 22 species of Fabaceae from the NCBI website were selected to study. *Papaver somniferum* was used as the outgroup. The reference sequences were compared by MAFFT software with the parameter of ‘–auto’ (Katoh and Standley 2013). Based on the aligned sequences, a phylogenetic tree was built with 1000 bootstrap replicates by IQ-TREE (version 1.6.12) (Lam-Tung et al. 2015). As shown in the phylogenetic tree (Figure 1), all species were clustered on the correct clades. Phylogenetic analysis showed that *E. tubulosa* clustered in a separated branch in the Fabaceae, which was the sister of *Lupinus* species. The new provided molecular data is helpful to illuminate the Fabaceae evolution.

**Figure 1.**
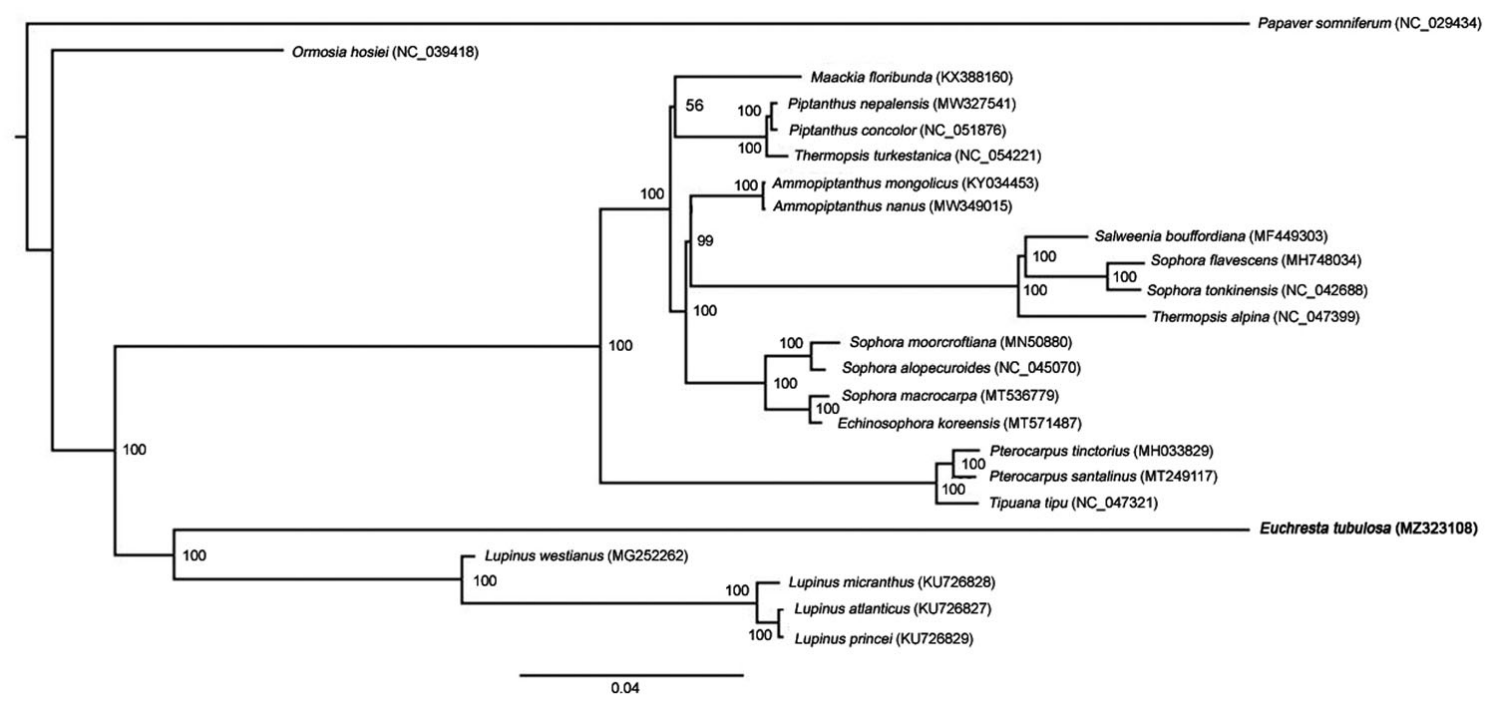
Maximum likelihood phylogenetic tree of *E. tubulosa* and other 22 Fabaceae species based on the complete chloroplast genome sequence. *Papaver somniferum* was used as the outgroup.

## Data Availability

The data that support the findings of this study are openly available in GenBank of NCBI at https://www.ncbi.nlm.nih.gov, under the accession number MZ323108. The associated Bio-Project, Bio-Sample and SRA numbers are PRJNA543381, SAMN19414659 and SRR14682996 respectively.
